# *Fructus mume* alleviates chronic cerebral hypoperfusion-induced white matter and hippocampal damage via inhibition of inflammation and downregulation of TLR4 and p38 MAPK signaling

**DOI:** 10.1186/s12906-015-0652-1

**Published:** 2015-04-22

**Authors:** Ki Mo Lee, JiHye Bang, Bu Yeo Kim, In Sun Lee, Jung-Soo Han, Bang Yeon Hwang, Won Kyung Jeon

**Affiliations:** KM-Based Herbal Drug Development Group, Korea Institute of Oriental Medicine, Daejeon, 305-811 Republic of Korea; Department of Biological Sciences, Konkuk University, 1 Hwayang-dong, Gwangjin-gu, Seoul, 143-701 Republic of Korea; College of Pharmacy, Chungbuk National University, Cheongju, 361-763 Republic of Korea

**Keywords:** Fructus mume, Permanent bilateral common carotid artery occlusion, Inflammation, White matter, Hippocampus

## Abstract

**Background:**

*Fructus mume* (*F. mume*) has been used as a traditional medicine for many years in Asian countries. The present study was designed to determine the effect of a 70% ethanol extract of *F. mume* on white matter and hippocampal damage induced by chronic cerebral hypoperfusion.

**Methods:**

Permanent bilateral common carotid artery occlusion (BCCAo) was performed on male Wistar rats to induce chronic cerebral hypoperfusion. Daily oral administration of *F. mume* (200 mg/kg) was initiated 21 days after BCCAo and continued for 42 days. The experimental groups in this study were divided into three groups: a sham-operated group, a BCCAo group, and a BCCAo group that was administered with the *F. mume* extract. The activation of glial cells, including microglia and astrocytes, and the levels of myelin basic protein (MBP), inflammatory mediators, Toll-like receptor 4 (TLR4), myeloid differentiation factor 88 (MyD88), and p38 mitogen-activated protein kinase (MAPK) phosphorylation were measured in brains from rats subjected to chronic BCCAo.

**Results:**

Our results revealed that *F. mume* alleviates the reduction in MBP expression caused by chronic BCCAo in the white matter and the hippocampus and significantly attenuates microglial and astrocytic activation induced by chronic BCCAo in the optic tract of white matter. In addition, *F. mume* treatment reduced the increased expression of cyclooxygenase-2 (COX-2), interleukin-1β (IL-1β) and interleukin-6 (IL-6), as well as the activation of TLR4/MyD88 and p38 MAPK signaling, in the hippocampus of rats subjected to chronic BCCAo.

**Conclusion:**

Taken together, our findings demonstrate that brain injury induced by chronic BCCAo is ameliorated by the anti-inflammatory effects of *F. mume* via inhibition of MBP degradation, microglial and astrocytic activation, increased inflammatory mediator expression, and activated intracellular signalings, including TLR4 and p38 MAPK, implying that *F. mume* is potentially an effective therapeutics for the treatment of vascular dementia.

**Electronic supplementary material:**

The online version of this article (doi:10.1186/s12906-015-0652-1) contains supplementary material, which is available to authorized users.

## Background

Cerebral blood flow (CBF) is tightly regulated by local brain metabolism [1]. Nevertheless, several vascular risk factors alter this homeostasis, consequently leading to the decrease in CBF. A long-lasting reduction in CBF, defined as chronic cerebral hypoperfusion, causes neurologic dysfunctions, including cognitive impairment and neuronal damage [2], and it is involved in the development of dementia such as Alzheimer’s disease (AD) and vascular dementia (VaD) [3-5]. The rat model of permanent bilateral common carotid artery occlusion (BCCAo) is widely used to examine mild chronic cerebral hypoperfusion [5,6]. BCCAo immediately decreases CBF after occlusion and maintains a persistent reduction in CBF for several months [7]. This reduction in CBF of rats subjected to BCCAo exhibits regional differences in various brain structures. For example, CBF decreased by 35-45% of the normal level in the cortex and the white matter and declined by 60% of the normal level in the hippocampus of BCCAo-induced rats [5]. Experimental evidences have demonstrated that chronic cerebral hypoperfusion induced by BCCAo results in damage to white matter regions, especially the corpus callosum and the optic tract, because these regions are more vulnerable to persistent obstruction of the blood supply from the carotid artery [8-10].

Neuroinflammation has been known to be correlated with the occurrence of white matter lesions induced by chronic cerebral hypoperfusion [11]. The inflammatory response leads to activation of resident glial cells, such as microglia and astrocytes, and upregulation of inflammatory cytokines, such as tumor necrosis factor-α (TNF-α) and interleukin-1β (IL-1β), aggravates the inflammation after chronic cerebral hypoperfusion [12]. *Fructus mume* (*F. mume*), the processed unripe fruit of Prunus mume, has been utilized for the treatment of gastrointestinal diseases in Asian countries for thousands of years. Several reports have demonstrated that *F. mume* is useful for the treatment of colitis in an inflammatory bowel disease model [13] and attenuates lipopolysaccharide-induced inflammation in RAW 264.7 cells by inhibiting the production of nitric oxide, prostaglandin E2, cyclooxygenase-2 (COX-2), and interleukin-6 (IL-6) [14]. However, the protective effect and mechanism of action of *F. mume* have not yet been fully investigated in various pathological conditions. Therefore, the aim of this study was to evaluate the effect of *F. mume* on the white matter and hippocampal damage in chronic cerebral hypoperfused rat and the potential efficacy of *F. mume* for the treatment of cognitive impairment.

## Methods

### Animals

Fifty-five male Wistar rats were used in the chronic BCCAo experiment (12 weeks old; Charles River Co., Gapyung, South Korea). For 2 weeks at the beginning of the experiment, the rats were housed in a vivarium at the Korea Institute of Oriental Medicine (KIOM) under controlled temperature (22 ± 1°C) and humidity (55 ± 10%) with a 12 h light/dark cycle (lights on at 08:00 h). Food and water were given *ad libitum* to all rats throughout the experiment. All experimental procedures described in this report were approved by the Institutional Animal Care and Use Committee of the KIOM.

### Surgery

The Wistar rats were anesthetized with 5% isoflurane in a mixture of 30% oxygen/70% nitrogen. Anesthesia was maintained with 3% isoflurane using a face mask during the surgical procedure. A midline incision was performed to expose both common carotid arteries, which were then tightly double-ligated using silk sutures. In addition, control rats were subjected to a sham operation in which they underwent the same procedure without BCCAo. Rectal temperature was maintained at 37.0 ± 0.5°C using a heating pad throughout the surgical procedure. After permanent BCCAo, two rats displayed neurological complications, such as seizures with squatting, and these rats died within one week after BCCAo surgery. In addition, three rats whose weight decreased to 20% of their weight prior to the surgery during drug or vehicle administration were excluded from this study.

### Preparation and administration of *F. Mume* extract

*F. mume*, produced in China, was obtained from a commercial supplier (Kwangmyung-Dang, Ulsan, Korea) in 2012. It was identified by the Herbal Quality Control Team and deposited at the Creative Research Laboratory of the KIOM. Dried *F. mume* was pulverized and extracted in 70% ethanol (EtOH) for 3 h at room temperature using an ultrasound-assisted extractor (OM30-EP; Sonimedi, Korea). The extract was concentrated under a vacuum using a rotary evaporator after filtration. The Wistar rats used in the present study were segregated into three groups: a sham-operated group that was orally administered the drug vehicle daily (n = 16); a BCCAo group that was orally administered the drug vehicle daily (n = 18); and a BCCAo group that was orally administered the *F. mume* extract 200 mg/kg once a day (n = 20). Vehicle or drug treatment was initiated on the 21th day after BCCAo or sham surgery and was continued until the 41st day after first vehicle/drug treatment by employing the oral gavage method. During oral administration, two rats were lost from *F. mume* extract treatment group due to the stress related to long-term oral feeding, but the *F. mume* extract displayed no toxicity with respect to changes in general behavior and mortality. The present study used normal saline as the vehicle.

### High performance liquid chromatography (HPLC) analysis of the *F. Mume* extract

HPLC was accomplished using a Waters Alliance e2695 HPLC system with a 2996 PDA detector and a YMC Hydrosphere C-18 column (5 mm, 4.6 × 250 mm i.d.) (YMC Co. Ltd., Tokyo, Japan). The mobile phase was comprised of acetonitrile (A) and water (B) using a stepwise gradient elution mixture: (A)/(B) = 0/100 (0 min), (A)/(B) = 10/90 (15 min), and (A)/(B) = 100/0 (40 min; hold for 10 min). The flow rate was 1.0 ml/min. The crude 70% EtOH extract of *F. mume* was filtered by membrane filters with a pore size 0.45 mm (Millipore), and the injection volume was 10 μl.

### Western blot analysis

After the administration of *F. mume* extract or vehicle was completed, all Wistar rats were decapitated, and their brains were micro dissected and then frozen. Protein samples of the hippocampal tissue were extracted in the following manner. Individual tissue samples were weighed and then homogenized in five volumes of ice-cold RIPA buffer containing 25 mM Tris HCl pH 7.6, 150 mM NaCl, 1% NP-40, 1% sodium deoxycholate, 0.1% SDS (Thermo Scientific, Waltham, MA, USA), protease inhibitor cocktail solution and phosphatase inhibitor cocktail solution (GenDEPOT, Barker, TX, USA). The homogenates were then centrifuged at 20,800 × g for 1 h at 4°C, and the supernatants were harvested, snap-frozen, and stored at −80°C. The protein concentration of the supernatants was determined using the BCA assay (Thermo Scientific, Waltham, MA, USA). Equal amounts of protein (40 μg) were then separated via SDS-PAGE and transferred to a PVDF membrane, which was subsequently incubated in a primary antibody (Ab) against COX-2 (Santa Cruz Biotechnologies, CA, USA), IL-1β (Millipore Corporation, MA, USA), IL-6 (Abcam, CA, USA), Toll-like receptor 4 (TLR4), myeloid differentiation factor 88 (MyD88, Santa Cruz Biotechnologies, CA, USA), phospho-p38 mitogen-activated protein kinase (MAPK), p38 MAPK (Cell Signaling, MA, USA), or glyceraldehyde-3-phosphate dehydrogenase (GAPDH, Santa Cruz Biotechnologies, CA, USA). Following incubation in the primary Ab, the membranes were incubated in horseradish peroxidase (HRP)-conjugated secondary Ab (Cell Signaling, MA, USA) and then detected using an ECL system (Thermo Scientific, MA, USA) with a Lumino Image Analyzer (Las-4000; Fujifilm, Tokyo, Japan). Densitometry was performed for specific markers normalized to GAPDH using Image J (v1.37) software.

### Immunohistochemistry

After the final administration of the *F. mume* extract or vehicle, all rats were euthanized using lethal overdose of Zoletil (50 mg/kg) and Rompun (5 mg/kg), followed by intracardial perfusion with 4% paraformaldehyde in 0.1 M phosphate buffer (pH 7.4). Following fixation, the brains were removed, post-fixed, treated with distilled water containing 30% sucrose for cryoprotection, snap-frozen, and sectioned (coronal plane: 40-μm) using a microtome. Next, the sections were blocked overnight at 4°C using 1% casein in phosphate buffered saline (PBS) containing 0.3% Triton-X 100. Then, the sections were incubated in antibody against NeuN (Millipore Corporation, MA, USA), myelin basic protein (MBP, Abcam, CA, USA), ionized calcium binding adaptor molecule-1 (Iba-1, Wako, Tokyo, Japan), or glial fibrillary acidic protein (GFAP, Abcam, CA, USA) for 1 h at room temperature. The sections were washed with PBS three times for 10 min each and then incubated in the appropriate biotinylated secondary antibodies (Thermo Scientific, MA, USA) for 2 h. Subsequently, the sections were incubated in the ExtrAvidin peroxidase conjugate (Sigma Aldrich, MO, USA) for 1 h. Finally, the stained sections were treated with a Vector SG substrate kit and a Vector DAB kit (Vector Laboratories, CA, USA) for peroxidase-mediated staining. Sections were then mounted onto resin-coated slides and dried for up to 1 week. They were finally coverslipped using permount reagent and images were taken at magnifications of 40× or 200× with an electron microscope (Olympus, Tokyo, Japan).

To quantify glial cells, the number of Iba-1- or GFAP-positive cells was counted. For a quantitative analysis, we selected specific regions reported to show neuroinflammatory changes [15]. Sections including the corpus callosum, fimbria, optic tract, and hippocampus from 7–9 rats per group were analyzed and Iba-1- and GFAP-positive cells were counted in ROIs of 0.03 mm^2^ in each region using the image J (v1.37) software. In addition, the optical density of MBP staining was measured in ROIs of 0.03 mm^2^ in each region using the Metamorph analysis software (Molecular devices, CA, USA). The observed number of Iba-1- and GFAP-positive cells, as well as the optical density of MBP staining in each ROI was averaged.

### Statistical analysis

One-way ANOVAs were performed to determine the effects of *F. mume* administration on alterations in the number of MBP, NeuN, GFAP, and Iba-1-positive cells, as well as the expression levels of COX-2, IL-1β, IL-6, TLR4, MyD88, and p38 MAPK induced by chronic BCCAo. Tukey’s post-hoc analyses were subsequently performed to determine the significance of the effects of *F. mume* treatment in chronic BCCAo rats. Unless otherwise specified, p values less than 0.05 were considered to be significant. All data are expressed as the mean ± SEM.

## Results

### HPLC analysis of 70% EtOH extract of *F. mume*

As shown in Additional file 1: Figure S1, the 70% EtOH extract of *F. mume* contained benzyl-O-β-D-glucopyranoside (1), benzyl-O-α-L-arabinopyranosyl-β-D-glucopyranoside (2), benzyl-O-β-D-xylopyranosyl-β-D-glucopyranoside (3), prunasin (4), α-hydroxy-benzeneacetamide (5), and 5-hydroxymethyl-2-furaldehyde (6).

### *F. mume* restored the MBP expression reduced by chronic cerebral hypoperfusion

**Figure 1:**
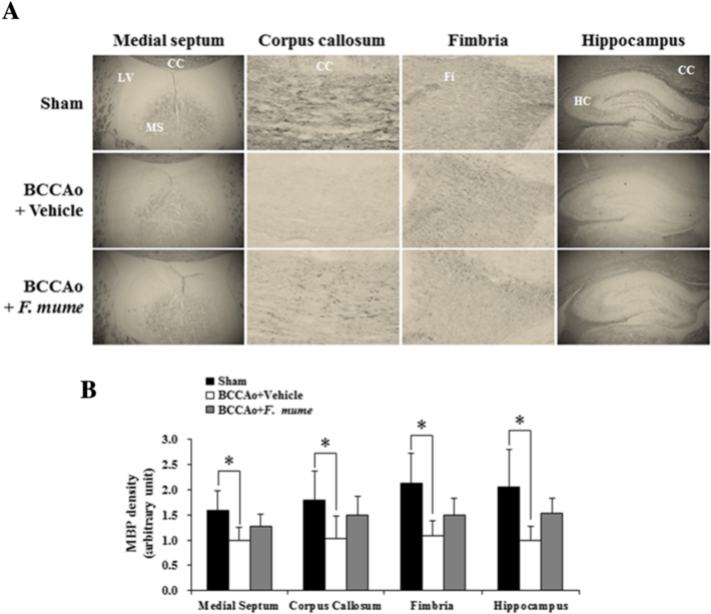
Effect of *F. mume* on MBP expression in the white matter and the hippocampus.. Immunohistological staining was performed to evaluate the expression level of MBP in the white matter regions and the hippocampus in the sham-operated group (n = 8), the BCCAo + Vehicle group (n = 9), and the BCCAo + *F. mume* group (n = 9). **(A)** Representative photomicrograph of MBP-positive cells. **(B)** MBP level was decreased in the medial septum, the corpus callosum, the fimbria of the white matter, and the hippocampus of BCCAo-injured rats compared to sham-operated rats (*). Data were analyzed via ANOVA followed by the Tukey test.*, p < 0.05 versus the BCCAo + Vehicle group. LV, lateral ventricle; MS, medial septum; CC, corpus callosum; FI, fimbria; HC, hippocampus; CC, corpus callosum.

The white matter is principally composed of myelin and nerve fibers. The degradation of myelin, an insulating layer that forms the myelin sheath, is a causative factor in white matter damage induced by chronic cerebral hypoperfusion, and MBP plays an important role in myelination as a component in the myelin sheath [16]. We investigated the effects of *F. mume* on chronic BCCAo-induced MBP breakdown in the medial septum, corpus callosum, and fimbria of the white matter and the hippocampus. ANOVA analysis revealed significant group effects of MBP expression in the medial septum, corpus callosum, and fimbria of the white matter and the hippocampus between sham-operated controls, BCCAo rats treated with vehicle, and BCCAo rats treated with *F. mume* (F(2,23) ≥ 5.94, p < 0.01). Post-hoc analyses revealed that compared to the sham-operated controls, the expression level of MBP in the BCCAo rats treated with vehicle was apparently decreased in the medial septum, corpus callosum, and fimbria of the white matter and the hippocampus (Figure 1A and B). *F. mume* restored the level of MBP expression that was reduced by BCCAo in these regions, but these effects of *F. mume* were not statistically significant.

### *F. mume* attenuated microglial and astrocytic activation induced by chronic cerebral hypoperfusion

**Figure 2:**
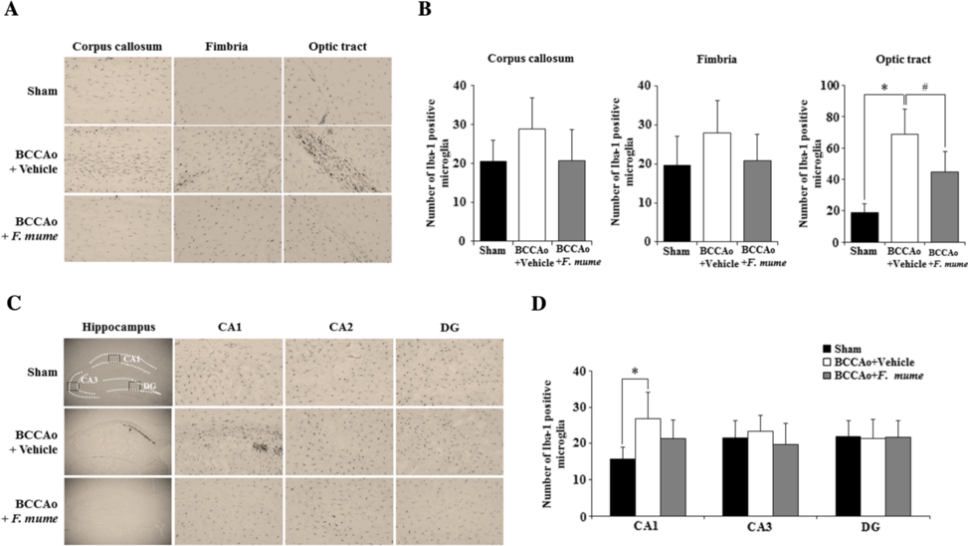
Effects of *F. mume* on chronic BCCAo-induced microglial activation in the white matter. Immunohistological staining was conducted to investigate the expression of Iba-1-positive cells in the white matter and the hippocampus in the sham-operated group (n = 7-8), the BCCAo + Vehicle group (n = 9), and the BCCAo + F. mume group (n = 9). Representative photomicrograph of Iba-1- **(A and B)**. The number of Iba-1- **(C and D)** positive cells was increased in the cerebral cortex, fimbria, the optic tract and CA1 of the BCCAo-injured rats compared to the sham-operated rats (*), and was significantly decreased in the optic tract of the BCCAo rats treated with *F. mume* compared to the BCCAo-injured rats (#). Data were analyzed via ANOVA followed by the Tukey test. *, p < 0.05 versus the BCCAo + Vehicle group; #, p < 0.05 versus the BCCAo + *F. mume* group. CA 1 and 3, cornuammonis 1 and 3; DG, dentate gyrus.

**Figure 3:**
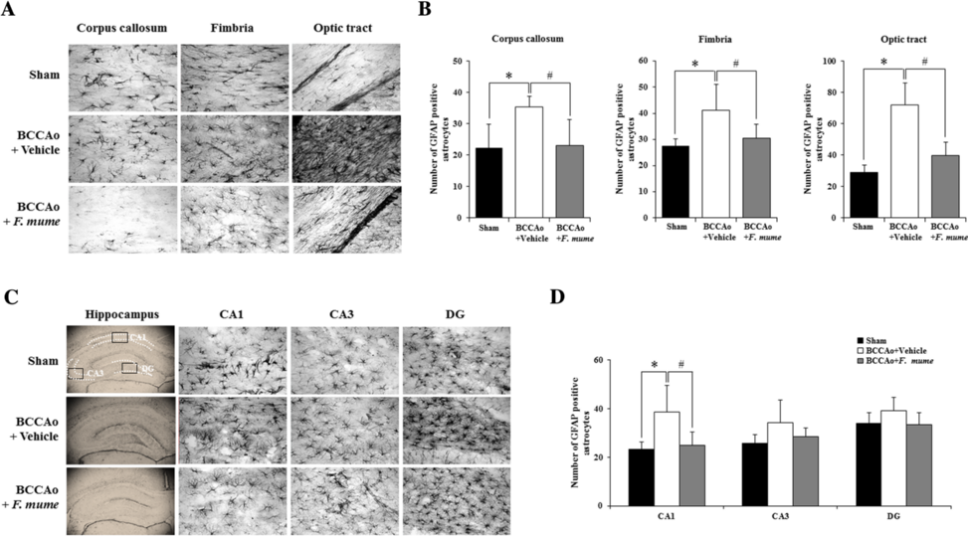
Effects of *F. mume* on chronic BCCAo-induced astrocytic activation in the white matter. Immunohistological staining was conducted to investigate the expression of GFAP-positive cells in the white matter and the hippocampus in the sham-operated group (n = 7-8), the BCCAo + Vehicle group (n = 9), and the BCCAo + F. mume group (n = 9). Representative photomicrograph of GFAP **(A and B)** positive cells. The number of GFAP **(C and D)**-positive cells was increased in the cerebral cortex, fimbria, the optic tract and CA1 of the BCCAo-injured rats compared to the sham-operated rats (*), and was significantly decreased in the optic tract of the BCCAo rats treated with *F. mume* compared to the BCCAo-injured rats (#). Data were analyzed via ANOVA followed by the Tukey test. *, p < 0.05 versus the BCCAo + Vehicle group; #, p < 0.05 versus the BCCAo + *F. mume* group. CA 1 and 3, cornuammonis 1 and 3; DG, dentate gyrus.

Glial cells, such as astrocytes and microglia, play a central role in neuroinflammation, and activation of these cells is detected in the brain of animals subjected to chronic cerebral hypoperfusion [17,18]. To investigate whether *F. mume* affects microglial and astrocytic activation in the white matter and the hippocampus of rats subjected to BCCAo, Iba-1 and GFAP are used as activation markers of microglia and astrocytes, respectively [19]. ANOVA analysis revealed meaningful group effects in the corpus callosum (Iba-1, F(2,23) = 3.84, p = 3.64e-02; GFAP, F(2,23) = 6.27, p = 2.40e-03), the fimbria (GFAP, F(2,22) = 7.47, p = 9.18e-04), and the optic tract (Iba-1, F(2,22) = 28.92, p = 6.95e-07; GFAP, F(2,22) = 26.7, p = 4.12e-08) of the white matter, and the CA1 (Iba-1, F(2,22) = 7.79, p = 2.78e-03; GFAP, F(2,22) = 8.50, p = 4.22e-04) of the hippocampus between sham-operated controls, BCCAo rats treated with vehicle, and BCCAo rats treated with *F. mume* (Figures 2 and 3). Post-hoc analyses indicated that compared to the sham-operated controls, the number of Iba-1-positive cells in the optic tract and the CA1 of the vehicle-treated BCCAo rats was remarkably increased (Figure 2B and D), and the number of GFAP-positive cells was also raised in the corpus callosum, the fimbria, the optic tract and the CA1 (Figure 3 from B to D). In addition, *F. mume* treatment significantly inhibited the increase in Iba-1-positive cells in the optic tract and in GFAP-positive cells in the corpus callosum, the fimbria, the optic tract and the CA1 compared to BCCAo rats treated with vehicle. In addition, the neuronal cell death induced by chronic BCCAo is reduced in the hippocampus of BCCAo rats treated with *F. mume*. However, this finding did not show statistical significance (Additional file 2: Figure S2).

### *F. mume* alleviated the increased expression of inflammatory mediators induced by chronic cerebral hypoperfusion

**Figure 4:**
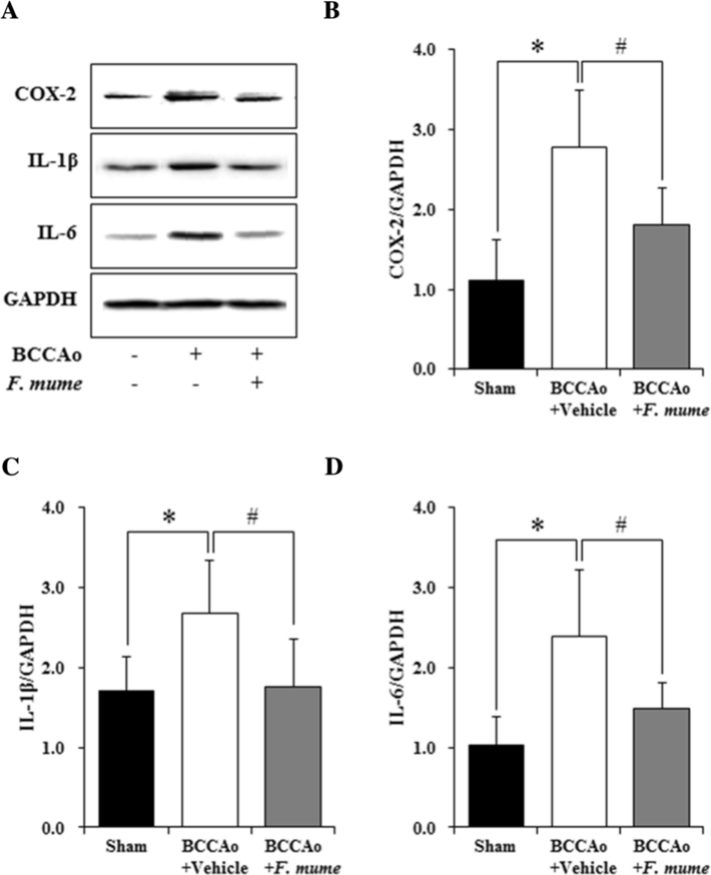
Effects of *F. mume* on hippocampal expression level of inflammatory mediators in chronic BCCAo rats. **(A)** Representative western blots of COX-2, IL-1β, and IL-6. Densitometry was performed for COX-2, IL-1β, and IL-6 normalized to GAPDH using Image J (v1.37) software. **(B through D)** The expression level of the inflammatory mediators (COX-2, IL-1β, and IL-6) was increased in the BCCAo + Vehicle group (n = 10) compared to the sham-operated group (n = 8). Treatment with *F. mume* (n = 8) attenuated the increased level of the inflammatory mediators induced by chronic BCCAo in the hippocampus (#). Data were analyzed via ANOVA followed by the Tukey test. *, p < 0.05 versus the BCCAo + Vehicle group; #, p <0.05 versus the BCCAo + *F. mume* group.

The inflammatory response has been hypothesized to cause the white matter and the hippocampal damage after chronic cerebral hypoperfusion [20]. Proinflammatory cytokines are released from activated microglia and astrocytes, which subsequently contributes to neuronal injury [21,22]. In the present study, we investigated the hippocampal level of COX-2, IL-1β, and IL-6 to determine the effect of *F. mume* treatment in chronic BCCAo rats. ANOVA analysis revealed significant group effects of the hippocampal level of COX-2, IL-1β, and IL-6 (F(2,23) ≥ 8.16, p < 0.01) between sham-operated controls, BCCAo rats treated with vehicle, and BCCAo rats treated with *F. mume*. Post-hoc analyses of the group effects indicated that chronic BCCAo rats exhibited the increase of COX-2, IL-1β, and IL-6 expression in the hippocampus (Figure 4A through D) compared to sham-operated control rats. The increased expression of these proinflammatory markers induced by chronic BCCAo was significantly decreased in chronic BCCAo rats treated with *F. mume* (Figure 4A through D).

### *F. mume* downregulated TLR4 and p38 MAPK signaling in the hippocampus

**Figure 5:**
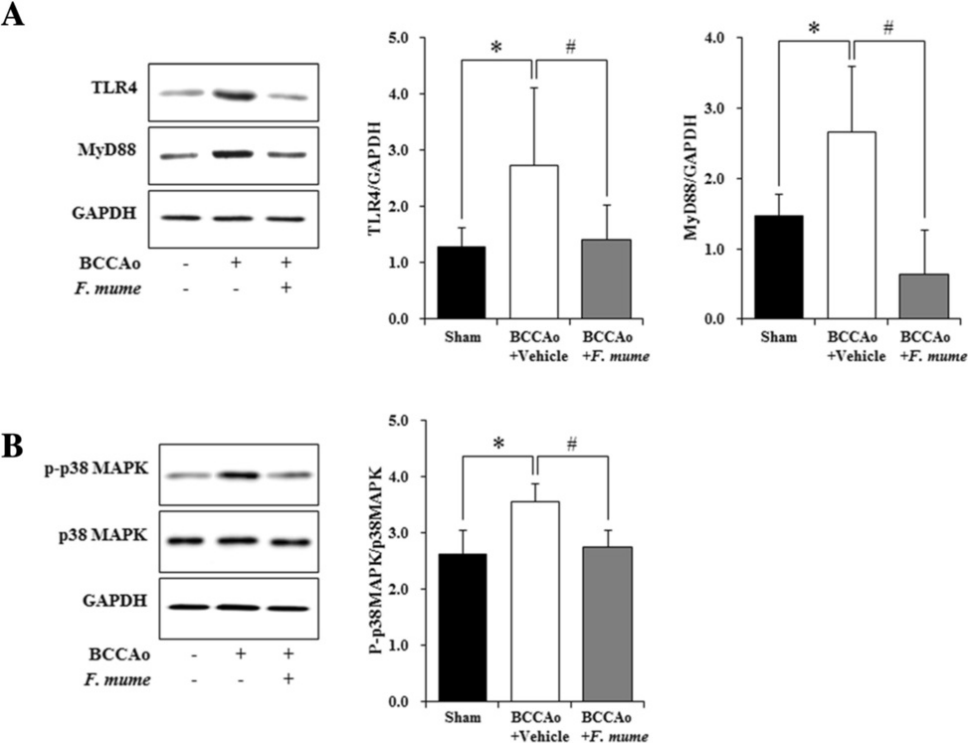
Effects of *F. mume* on the activation of TLR4 and p38 MAPK signaling. (A and B) Representative western blots of TLR4, MyD88, phospho-p38 MAPK, p38 MAPK, and GAPDH. Densitometry was performed for TLR4, MyD88, phospho-p38 MAPK, and p38 MAPK normalized to GAPDH using Image J (v1.37) software. **(A and B)** The hippocampal levels of TLR4, MyD88, and phospho-p38 MAPK were increased in the BCCAo + Vehicle group (n = 10) (*) compared to the sham-operated group (n = 8). Treatment with *F. mume* (n = 8) decreased the hippocampal levels of TLR4, MyD88, and phospho-p38 MAPK increased by chronic BCCAo (#). Data were analyzed via ANOVA followed by the Tukey test. *, p < 0.05 versus the BCCAo + Vehicle group; #, p <0.05 versus the BCCAo + *F. mume* group.

TLR4 signaling is involved in both glial cell activation and upregulation of proinflammatory cytokines [23-25]. To explore the effect of *F. mume* on TLR4 signaling in chronic BCCAo rats, we measured the hippocampal level of TLR4 and MyD88. ANOVA analysis of TLR4 and MyD88 expression revealed significant group effects in the hippocampus between sham operated controls, BCCAo rats treated with vehicle, and BCCAo rats treated with *F. mume* (F(2,23) ≥ 6.52, p < 0.01). Subsequent post-hoc analyses revealed that the expression level of TLR4 and MyD88 was increased in the hippocampus of chronic BCCAo rats treated withvehicle compared to sham-operated rats (Figure 5A and B). The increased hippocampal level of TLR4 and MyD88 induced by chronic BCCAo was not observed in BCCAo rats treated with *F. mume* (Figure 5A). MAPK signaling is associated with the production of proinflammatory mediators in microglia and astrocytes [20] and is activated in the brains of rats subjected to chronic BCCAo [26-29]. We examined the correlation between *F. mume* treatment and p38 MAPK phosphorylation in the hippocampus in rats subjected to chronic BCCAo. ANOVA analysis of p38 MAPK phosphorylation revealed significant group effects in the hippocampus (F(2,23) = 19.93, p = 9.52e-06). Subsequent post-hoc analyses indicated that the level of p38 MAPK phosphorylation was increased in the hippocampus of chronic BCCAo rats treated with vehicle compared to sham-operated rats (Figure 5B). The increase in the hippocampal level of p38 MAPK phosphorylation induced by chronic BCCAo was significantly reduced in chronic BCCAo rats treated with *F. mume* (Figure 5B). These findings suggest that the anti-inflammatory effects of *F. mume* might be mediated via inhibition of the activation of TLR4 and p38 MAPK signaling in response to chronic cerebral hypoperfusion.

## Discussion

In a previous study, we demonstrated that *F. mume* extracted using hot water mitigates the spatial memory impairments induced by chronic BCCAo via the restoration of abnormal microglial activation and altered signaling of extracellular signal-regulated kinases (ERKs) or nuclear factor-κB (NF-κB) in the hippocampus [26]. Our present study demonstrated a neuroprotective effect of *F. mume* extracted using 70% EtOH on chronic cerebral hypoperfusion-induced brain dysfunction through inhibiting MBP degradation, activation of microglia and astrocytes, increased proinflammatory mediator expression, and stimulation of TLR4 and p38 MAPK signaling. *F. mume* has long been used as a traditional medicine in Asian contries. The efficacy of *F. mume* has been known to include anti-bacterial [30], anti-inflammatory [14], and anti-diabetic properties [31]. According to HPLC analysis, the 70% EtOH extract of *F. mume* had benzyl-O-β-D-glucopyranoside, benzyl-O-α-L-arabinopyranosyl-β-D-glycopyranoside, benzyl-O-β-D-xylopyranosyl-β-D-glycopyranoside, prunasin, α-hydroxy-benzeneacetamide, and 5-hydroxymethyl-2-furaldehyde (Additional file 1: Figure S1).

White matter, which is generally composed of glial cells and myelinated axons, is vulnerable to chronic cerebral hypoperfusion [32]. White matter lesions are detected in the brains of AD and VaD patients with chronic cerebral hypoperfusion as a result of glial cell activation and myelin sheath degradation [5]. Also, to study the relationship between demyelination and inflammation in the injured brain after hypoxia-ischemia, MBP expression and glial cell activation have been measured, since demyelination was accompanied with neuroinflammtion, such as activation of glial cells and release of proinflammatory cytokines [33,34].

In the present study, MBP expression, a marker of myelin sheath structure, was significantly decreased in the white matter of rats subjected to chronic BCCAo, but this reduction was alleviated by administration of *F. mume*. This result indicates that *F. mume* can protect the brain from chronic cerebral hypoperfusion-induced white matter lesions by restoring the decrease in MBP expression. Inflammatory events play a crucial role in the pathogenesis of neurological diseases, including AD and VaD [35]. Chronic cerebral hypoperfusion-induced inflammation is characterized by activation of glial cells and overexpression of inflammatory mediators, such as TNF-α and IL-1β [8,11,36]. Therefore, inhibition of these inflammatory responses is important for protecting the brain injury induced by chronic cerebral hypoperfusion. Microglia and astrocytes are activated by various events, such as ischemia, infection, and inflammation, which subsequently stimulate the release of cytotoxic agents, including cytokines, matrix metallo proteinases, nitric oxide, and reactive oxygen species [12]. We found that *F. mume* significantly attenuates microglial and astrocytic activation in the white matter and hippocampus in rats subjected to chronic BCCAo. In addition, treatment with *F. mume* alleviates the increase in hippocampal COX-2, IL-1β, and IL-6 expression in chronic BCCAo rats. Toll-like receptors (TLRs) play a pivotal role in the initiation of innate immune responses. Among the identified TLRs, TLR4 is expressed on microglia and astrocytes and participates in activation of these cells induced by hypoxia in the brain [22,37,38]. Several studies have reported that TLR4-dependent activation of microglia and astrocytes is involved in neurodegenerative diseases [35,38]. We have previously demonstrated that *F. mume* inhibits the stimulation of IKK/NF-κB signaling induced by chronic BCCAo in the rat hippocampus [26]. Moreover, the phosphorylation of MAPK-related signaling molecules, such as ERKs, c-Jun amino-terminal kinases (JNKs), and p38 MAPK, is important for the production of proinflammatory mediators, including TNF-α, IL-1β, and IL-6, in microglia and astrocytes [20,28]. It has been reported that specific inhibition of p38 MAPK using SB203580 attenuated the increase in expression of TNF-α, IL-1β, and IL-6 in the hippocampus activated by mitogenic factor-induced inflammation [39]. In the present study, we demonstrated that activation of TLR4/MyD88 signaling and p38 MAPK signaling is suppressed by treatment with *F. mume* in rats subjected to chronic BCCAo.

## Conclusion

The present study demonstrated that *F. mume* exerts an anti-inflammatory effect by attenuating white matter lesions, microglial and astrocytic activation, the increased expression of proinflammatory mediators, and the stimulation of TLR4 and p38 MAPK signaling that are induced by chronic cerebral hypoperfusion, suggesting a novel mechanism to explain the anti-inflammatory action of *F. mume* and its potential usefulness as a neuroprotective agent for the treatment and prevention of neurodegenerative diseases.

## Additional files

Additional file 1: Figure S1. HPLC chromatogram of the 70% EtOH extract ofF. mume. Benzyl-O-β-D-glucopyranoside (1), benzyl-O-α-L-arabinopyranosyl-β-D-glucopyranoside (2), benzyl-O-β-D-xylopyranosyl-β-D-glucopyranoside (3), prunasin (4), α-hydroxy-benzeneacetamide (5), and 5-hydroxymethyl-2-furaldehyde (6). Additional file 2: Figure S2. Effect of F. mumeon chronic BCCAo-induced neuronal cell death in the hippocampus. Immunohistological staining was performed to determine the number of NeuN-positive cells in CA1, CA3 and DG regions of hippocampus in the sham-operated group (n = 8), BCCAo + Vehicle group (n = 9), and BCCAo + *F. mume* group (n = 9). (A) Representative photomicrograph of NeuN-positive cells. To quantify neuronal cell death, the number of NeuN positive cells was counted in CA1, CA3 and DG regions of hippocampus. One region of interest (ROI) of 0.03 mm^2^ per one section in CA1, CA3, and DG of hippocampus were selected. The number of NeuN positive cells was counted in each ROI and averaged. (B) NeuN-positive cells were reduced in CA1, CA3, and DG of the hippocampus in the chronic BCCAo rats compared to sham-operated control rats. The reduction of NeuN-positive cells induced by chronic BCCAo was not observed in chronic BCCAo rats treated with *F. mume*. However, the statistical significance in these results was not observed. CA 1 and 3, cornu ammonis 1 and 3; DG, dentate gyrus.

## References

[1] Gurol ME (2013). Cerebral hypoperfusion and white matter disease in healthy elderly and patients with Alzheimer’s disease. Eur J Neurol.

[2] O’Sullivan M (2008). Leukoaraiosis. PractNeurol.

[3] de la Torre JC (2000). Critically attained threshold of cerebral hypoperfusion: the CATCH hypothesis of Alzheimer’s pathogenesis. Neurobiol Aging.

[4] Roman GC, Erkinjuntti T, Wallin A, Pantoni L, Chui HC (2002). Subcortical ischaemic vascular dementia. Lancet Neurol.

[5] Farkas E, Luiten PG, Bari F (2007). Permanent, bilateral common carotid artery occlusion in the rat: a model for chronic cerebral hypoperfusion-related neurodegenerative diseases. Brain Res Rev.

[6] Bang J, Jeon WK, Lee IS, Han JS, Kim BY (2013). Biphasic Functional Regulation in Hippocampus of Rat with Chronic Cerebral Hypoperfusion Induced by Permanent Occlusion of Bilateral Common Carotid Artery. PLoS One.

[7] Marosi M, Rakos G, Robotka H, Nemeth H, Sas K, Kis Z, Farkas T, Lur G, Vecsei L, Toldi J (2006). Hippocampal (CA1) activities in Wistar rats from different vendors. Fundamental differences in acute ischemia. J Neurosci Methods.

[8] Farkas E, Donka G, de Vos RAI, Mihaly A, Bari F, Luiten PGM (2004). Experimental cerebral hypoperfusion induces white matter injury and microglial activation in the rat brain. Acta Neuropathol.

[9] Ohta H, Nishikawa H, Kimura H, Anayama H, Miyamoto M (1997). Chronic cerebral hypoperfusion by permanent internal carotid ligation produces learningimpairment without brain damage in rats. Neuroscience.

[10] Wakita H, Tomimoto H, Akiguchi I, Matsuo A, Lin JX, Ihara M, McGeer PL (2002). Axonal damage and demyelination in the white matter after chronic cerebral hypoperfusion in the rat. Brain Res.

[11] Wakita H, Tomimoto H, Akiguchi I, Lin JX, Miyamoto K, Oka N (1999). A cyclooxygenase-2 inhibitor attenuates white matter damage in chronic cerebral ischemia. Neuroreport.

[12] Wang Q, Tang XN, Yenari MA (2007). The inflammatory response in stroke. J Neuroimmunol.

[13] Liu L, Yuan S, Sun Y, Long Y, Li Y, Niu Y, Li C, Gan H, Cao S, Mei Q (2009). The possible mechanisms of *Fructus mume* pill in the treatment of colitis induced by 2,4,6-trinitrobenzene sulfonic acid in rats. J Ethnopharmacol.

[14] Choi HJ, Kang OH, Park PS, Chae HS, Oh YC, Lee YS, Choi JG, Lee GH, Kweon OH, Kwon DY (2007). Mume Fructus water extract inhibits pro-inflammatory mediators in lipopolysaccharide-stimulated macrophages. J Med Food.

[15] Choi BR, Lee SR, Han JS, Woo SK, Kim KM, Choi DH, Kwon KJ, Han SH, Shin CY, Lee J, Chung CS, Lee SR, Kim HY (2011). Synergistic memory impairment through the interaction of chronic cerebral hypoperfusion and amlyloid toxicity in a rat model. Stroke.

[16] Walker EJ, Rosenberg GA (2010). Divergent role for MMP-2 in myelin breakdown and oligodendrocyte death following transient global ischemia. J Neurosci Res.

[17] Choi BR, Kwon KJ, Park SH, Jeon WK, Han SH, Kim HY, Han JS (2011). Alternations of Septal-hippocampal System in the Adult Wistar Rat with Spatial Memory Impairments Induced by Chronic Cerebral Hypoperfusion. Exp Neurobiol.

[18] Shibata M, Ohtani R, Ihara M, Tomimoto H (2004). White matter lesions and glial activation in a novel mouse model of chronic cerebral hypoperfusion. Stroke.

[19] Watanabe T, Zhang N, Liu M, Tanaka R, Mizuno Y, Urabe T (2006). Cilostazol protects against brain white matter damage and cognitive impairment in a rat model of chronic cerebral hypoperfusion. Stroke.

[20] Guo RB, Wang GF, Zhao AP, Gu J, Sun XL, Hu G (2012). Paeoniflorin protects againstischemia-induced brain damages in rats via inhibiting MAPKs/NF-kappaB-mediated inflammatory responses. PLoS One.

[21] Rubio-Perez JM, Morillas-Ruiz JM (2012). A review: inflammatory process in Alzheimer’s disease, role of cytokines. ScientificWorldJournal.

[22] Smith JA, Das A, Ray SK, Banik NL (2012). Role of pro-inflammatory cytokines released from microglia in neurodegenerative diseases. Brain Res Bull.

[23] Arroyo DS, Soria JA, Gaviglio EA, Rodriguez-Galan MC, Iribarren P (2011). Toll-like receptors are key players in neurodegeneration. Int Immunopharmacol.

[24] Jin JJ, Kim HD, Maxwell JA, Li L, Fukuchi K (2008). Toll-like receptor 4-dependent upregulation of cytokines in a transgenic mouse model of Alzheimer’s disease. J Neuroinflammation.

[25] Song M, Jin J, Lim JE, Kou J, Pattanayak A, Rehman JA, Kim HD, Tahara K, Lalonde R, Fukuchi K (2011). TLR4 mutation reduces microglial activation, increases Abeta deposits and exacerbates cognitive deficits in a mouse model of Alzheimer’s disease. J Neuroinflammation.

[26] Jeon WK, Ma J, Choi BR, Han SH, Jin Q, Hwang BY, Han JS (2012). Effects of *Fructusmume* Extract on MAPK and NF-kappaB Signaling and the Resultant Improvement in the Cognitive Deficits Induced by Chronic Cerebral Hypoperfusion. Evid Based Complement Alternat Med.

[27] Jung HW, Yoon CH, Park KM, Han HS, Park YK (2009). Hexane fraction of ZingiberisRhizomaCrudus extract inhibits the production of nitric oxide and proinflammatory cytokines in LPS-stimulated BV2 microglial cells via the NF-kappaB pathway. Food ChemToxicol.

[28] Pan XD, Chen XC, Zhu YG, Chen LM, Zhang J, Huang TW, Ye QY, Huang HP (2009). Tripchlorolide protects neuronal cells from microglia-mediated beta-amyloid neurotoxicity through inhibiting NF-kappaB and JNK signaling. Glia.

[29] Lee KM, Bang JH, Han JS, Kim BY, Lee IS, Kang HW, Jeon WK (2013). Cardiotonic pill attenuates white matter and hippocampal damage via inhibiting microglial activation and downregulating ERK and p38 MAPK signaling in chronic cerebral hypoperfused rat. BMC Complement Altern Med.

[30] Chen Y, Wong RW, Seneviratne CJ, Hagg U, McGrath C, Samaranayake LP, Kao R (2011). The antimicrobial efficacy of *Fructus mume* extract on orthodontic bracket: a monospecies-biofilm model study in vitro. Arch Oral Biol.

[31] Tu X, Xie C, Wang F, Chen Q, Zuo Z, Zhang Q, Wang X, Zhong S, Jordan JB (2013). *Fructusmume* formula in the treatment of type 2 diabetes mellitus: a randomized controlled pilot trial. Evid Based Complement Alternat Med.

[32] Yoshioka H, Niizuma K, Katsu M, Sakata H, Okami N, Chan PH (2011). Consistent injury tomedium spiny neurons and white matter in the mouse striatum after prolonged transient global cerebral ischemia. J Neurotrauma.

[33] Schmitz T, Chew LJ (2008). Cytokines and myelination in the central nervous system. Scientific World Journal.

[34] Li A, Lv S, Yu Z, Zhang Y, Ma H, Zhao H, Piao H, Li S, Zhang N, Sun C (2010). Simvastatin attenuates hypomyelination induced by hypoxia-ischemia in neonatal rats. Neurol Res.

[35] Trudler D, Farfara D, Frenkel D. Toll-like receptors expression and signaling inglia cells in neuro-amyloidogenic diseases: towards future therapeutic application. Mediators Inflamm. 2010;2010. doi:10.1155/2010/497987. 10.1155/2010/497987 PMC2913815 20706642

[36] Orzylowska O, Oderfeld-Nowak B, Zaremba M, Januszewski S, Mossakowski M (1999). Prolonged and concomitant induction of astroglialimmunoreactivity of interleukin-1beta and interleukin-6 in the rat hippocampus after transient global ischemia. Neurosci Lett.

[37] Yao L, Kan EM, Lu J, Hao A, Dheen ST, Kaur C, Ling EA (2013). Toll-like receptor 4 mediates microglial activation and production of inflammatory mediators in neonatal rat brain following hypoxia: role of TLR4 in hypoxic microglia. J Neuroinflammation.

[38] Zeng KW, Zhang T, Fu H, Liu GX, Wang XM (2012). Schisandrin B exerts anti-neuroinflammatory activity by inhibiting the Toll-like receptor 4-dependent MyD88/IKK/NF-KappaB signaling pathway in lipopolysaccharide-induced microglia. Eur J Pharmacol.

[39] Chaparro-Hyerta V, Rivera-Cervantes MC, Flores-Soto ME, Gomez-Pinedo U, Beas-Zarate C (2005). Prinflammatory cytokines and apoptosis following glutamate-induced excitotoxicity mediated by p38 MAPK in the hippocampus of neonatal rats. J Neuroimmunol.

