# Supplementary figures and images for: Fructus mume alleviates chronic cerebral hypoperfusion-induced white matter and hippocampal damage via inhibition of inflammation and downregulation of TLR4 and p38 MAPK signaling
Source: BMC Complement Altern Med. 2015 Apr 22;15:125. doi: 10.1186/s12906-015-0652-1 (PMC4411748; doi:10.1186/s12906-015-0652-1)

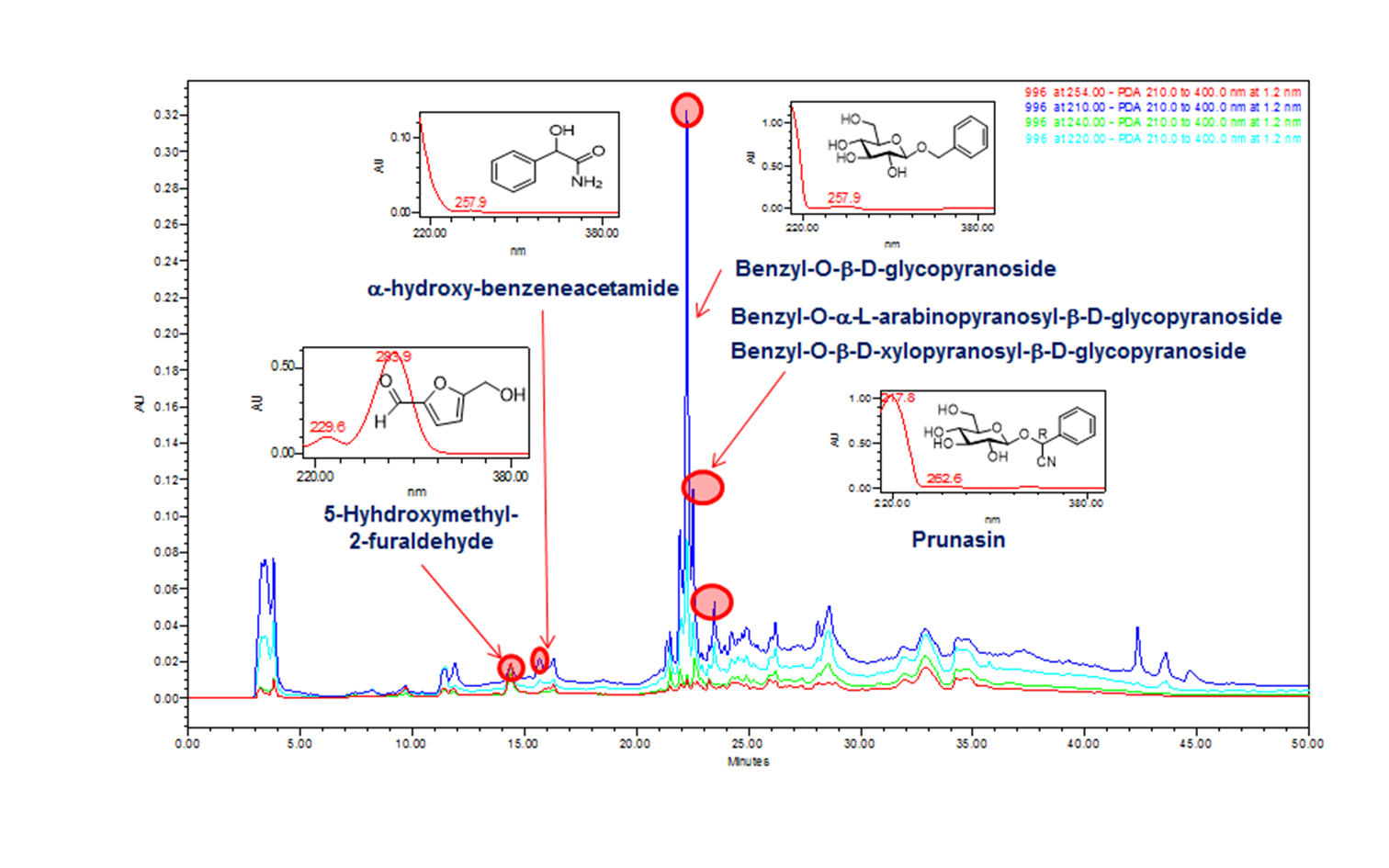

Supplement: Additional file 1: Figure S1. — HPLC chromatogram of the 70% EtOH extract ofF. mume. Benzyl-O-β-D-glucopyranoside (1), benzyl-O-α-L-arabinopyranosyl-β-D-glucopyranoside (2), benzyl-O-β-D-xylopyranosyl-β-D-glucopyranoside (3), prunasin (4), α-hydroxy-benzeneacetamide (5), and 5-hydroxymethyl-2-furaldehyde (6). [file 12906_2015_652_MOESM1_ESM.tiff]

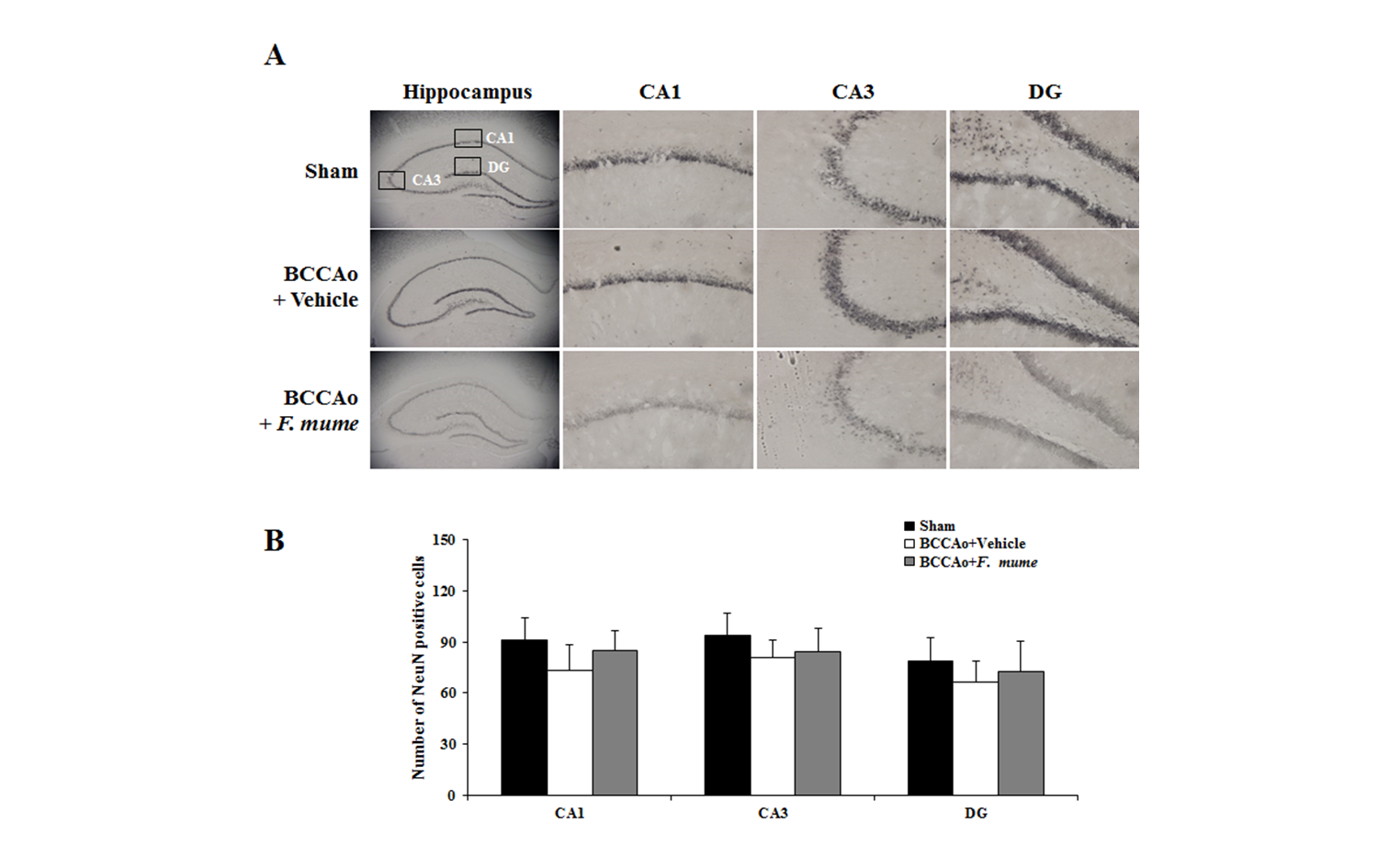

Supplement: Additional file 2: Figure S2. — Effect of F. mumeon chronic BCCAo-induced neuronal cell death in the hippocampus. Immunohistological staining was performed to determine the number of NeuN-positive cells in CA1, CA3 and DG regions of hippocampus in the sham-operated group (n = 8), BCCAo + Vehicle group (n = 9), and BCCAo + F. mume group (n = 9). (A) Representative photomicrograph of NeuN-positive cells. To quantify neuronal cell death, the number of NeuN positive cells was counted in CA1, CA3 and DG regions of hippocampus. One region of interest (ROI) of 0.03 mm2 per one section in CA1, CA3, and DG of hippocampus were selected. The number of NeuN positive cells was counted in each ROI and averaged. (B) NeuN-positive cells were reduced in CA1, CA3, and DG of the hippocampus in the chronic BCCAo rats compared to sham-operated control rats. The reduction of NeuN-positive cells induced by chronic BCCAo was not observed in chronic BCCAo rats treated with F. mume. However, the statistical significance in these results was not observed. CA 1 and 3, cornu ammonis 1 and 3; DG, dentate gyrus. [file 12906_2015_652_MOESM2_ESM.tiff]
